# Antiviral activity of the water extract and ethanol extract of *Sorbus commixta* against influenza A virus *in vitro*

**DOI:** 10.1016/j.heliyon.2024.e39049

**Published:** 2024-10-09

**Authors:** Yewon Kim, Sanghyun Lee, Chulwoo Kim, Sun-Woo Yoon, Sejin Jeon, Mi-Na Kweon, Baik-Lin Seong, Sang-Uk Seo, Yo Han Jang

**Affiliations:** a Department of Vaccine Biotechnology, Andong National University, Andong, Republic of Korea; b Department of Plant Science and Technology, Chung-Ang University, Anseong, Republic of Korea; c Department of Microbiology, Institute for Viral Diseases, Korea University College of Medicine, Seoul, Republic of Korea; d Department of Convergence Medicine, University of Ulsan College of Medicine, Seoul, Republic of Korea; e Department of Microbiology and Immunology, College of Medicine, Yonsei University, Seoul, Republic of Korea; f Department of Microbiology, College of Medicine, The Catholic University of Korea, Seoul, Republic of Korea

**Keywords:** Antiviral agents, Influenza virus, *Sorbus commixta*, natural compounds, Plant extracts

## Abstract

Although vaccines and antivirals are currently available, influenza virus infections continually present major threats to human health. Due to genetic diversity and variability of influenza viruses, the development of new antiviral agents against the virus remains as a considerable challenge. In this study, we evaluated the antiviral activity of the water extract and ethanol extract of *Sorbus commixta* Hedl. (SCH), widely used as a medical herb, against influenza A viruses and identified molecular mechanisms for the antiviral activity. The water extract and ethanol extract of SCH demonstrated virucidal activity against influenza A virus at the noncytotoxic concentrations. In addition, cytopathic effect reduction assay and GFP fluorescence image analysis suggest that SCH extracts have inhibitory activity on multiple stages during influenza virus life cycle. Mechanisms studies showed that SCH extracts inhibited the biological functions of influenza viral surface hemagglutinin and neuraminidase proteins that play critical roles in the viral entry and release steps. SCH extracts not only prevented the receptor binding of influenza viral HA to the cellular receptors but also inhibited the HA-mediated membrane fusion. In addition, SCH extracts suppressed the enzyme activity of the viral neuraminidase. The results together suggest that SCH extracts contain antiviral natural compounds that inhibit multiple influenza viral proteins including the viral surface proteins. Our findings suggest that SCH extracts could be promising resources for the development of novel antiviral agents against influenza A viruses.

## Highlights

• The antiviral activity of SCH extracts against influenza A virus was examined.
• SCH extracts showed virucidal activity against influenza virus.
• SCH extracts inhibited the biological functions of viral HA and NA proteins.
• SCH extracts inhibited the viral entry and release steps of influenza virus.
• SCH extracts provide a promising resource for antiviral agents to influenza viruses.

## 1 Introduction

Influenza viruses belong to *Orthomyxoviridae* family possessing seven or eight segmented negative-sense RNA genome. Influenza viruses are classified into four types (A, B, C, and D) based on the antigenicity of the nucleoprotein and matrix genes, with only type A and B viruses known to cause epidemic diseases in humans [1]. According to World Health Organization, it is estimated that seasonal influenza causes 3–5 million cases of severe illness and 290,000 to 650,000 deaths annually [2]. Due to segmented nature of RNA genome, influenza viruses demonstrate a high propensity for antigenic changes by antigenic shift and antigenic drift [3,4]. Influenza A viruses are subdivided into a number of subtypes according to the viral surface proteins, hemagglutinin (HA) and neuraminidase (NA). Thus far, 18 different HA subtypes and 11 different NA subtypes have been discovered, with more than 130 influenza A subtypes identified in nature [5]. The antigenic variability and diversity of influenza A viruses present great challenges for preventing the viral infections and controlling the epidemics and occasional pandemics [6,7]. In particular, sudden outbreak of a pandemic with a novel genetic reassortant virus poses a global problem, imposing medical and economic burdens due to the lack of a matched vaccine [8,9].

There are currently three classes of antivirals for treatment for influenza A virus infections approved for clinical uses, including M2 ion channel blockers, viral NA inhibitors, and viral RNA polymerase inhibitors [10]. However, increasing emergence of drug-resistant strains and drug-related side effects present significant challenges in clinical use [11,12], making continuous research for the development of new antiviral drugs essential. Plant-derived natural products are reported to have various health beneficial activities, including antioxidant, anticancer, and anti-inflammatory. In addition, increasing number of studies have shown that natural products from plants have antiviral potentials against diverse viruses [13,14], suggesting that natural products from plants can serve as a valuable source for developing new antiviral agents. *Sorbus commixta* Hedl. (SCH) have been reported to have anti-inflammatory, anti-cancer, anti-oxidative, and other various medicinal properties [15,16]. However, there are very limited reports on the antiviral activity of SCH. Only a study reported the antiviral activity of sakuranetin, a natural compound isolated from SCH, against human rhinovirus 3 [17]. However, the antiviral activity of SCH against influenza viruses have not been reported yet. In this study, we have shown that the water extract and ethanol extract of SCH possess potent antiviral activity against influenza A viruses. SCH extracts demonstrated not only virucidal activity but also inhibitory activity against the biological functions of influenza viral surface proteins. Our results merit further studies to identify individual natural compounds possessing antiviral activities for developing novel antiviral drug candidates against influenza A viruses.

## 2 Materials and methods

### 2.1 Cell lines and influenza A viruses

Madin-Darby canine kidney (MDCK) cells were purchased from American Type Culture Collection (ATCC) and were maintained in Minimum Essential Medium (Gibco, NY, USA) supplemented with 10 % fetal bovine serum (HyClone, Utah, USA) in 5 % CO_2_ incubator. Three strains of influenza A virus, A/Puerto Rico/8/34 (A/PR8, H1N1), A/PR8-GFP reporter virus [18], and mouse-adapted A/aquatic bird/Korea/w81/05 (A/MA81, H5N2), were propagated in MDCK cells. To prepare viral stocks, MDCK cells were infected with the virus and the supernatants were clarified by centrifugation and were aliquoted for storage at −80 °C until use.

### 2.2 Preparation of the water extract and ethanol extract of SCH

The raw materials of the branches and stem mixtures of SCH were purchased from Omniherb (Republic of Korea). To prepare water extract, dried raw materials were extracted with distilled water and lyophilized. To prepare ethanol extract, dried raw materials were extracted with ethanol three times under reflux and evaporated *in vacuo*. The water extracts were dissolved in PBS and the ethanol extracts were dissolved in DMSO.

### 2.3 Measurement of total polyphenols and antioxidative activity of SCH extracts

Total amounts of polyphenols contained in SCH extracts were measured using the Folin-Ciocalteu reagent, with gallic acid as the standard. 10 μl of the extracts and 200 μl of 2 % Na_2_CO_3_ were incubated for 3 min, and 10 μl of 50 % Folin reagent was added for additional incubation for 30 min, and the absorbance of the mixtures was measured at 760 nm. Total amounts of polyphenols were determined as gallic acid equivalents. Antioxidative activity of the extracts were measured by DPPH (2,2-diphenyl-1-picryl hydrazyl) radical scavenging assay, as previously described [19]. Briefly, 20 μl of the extracts were incubated with 180 μl of 100 μM DPPH reagent for 30 min, and the absorbance of the mixture was measured at 514 nm.

### 2.4 *In vitro* cytotoxicity tests of SCH extracts

*In vitro* cytotoxicity of the extracts was measured by Cell Counting Kit (CCK-8) (Dojindo Laboratories, Japan). MDCK cells were seeded in 96-well plates (10^4^ cells/well) and cultured for 24 h. Two-fold serial dilutions of each extract were treated to the cells for 24 h at 37 °C, with PBS and DMSO treated to the cells as vehicle controls. After the incubation, 10 μl of CCK-8 reagent was added to each well and incubated for 1 h at 37 °C. Absorbance of each well was measured at 450 nm was using a microplate reader. Cell viability (%) was calculated as [(absorbance of the extracts well – blank well absorbance)/(vehicle well absorbance-blank well absorbance)] × 100 %. The extract concentrations that yielded cell viability greater than 80 % compared to that of the control were considered as noncytotoxic, at which the extracts were treated to influenza viruses to examine the virucidal activity.

### 2.5 Hemagglutination inhibition assay

Hemagglutination assay was performed to measure the HA units of influenza A viruses. In V-bottom 96-well plates, two-fold serial dilutions of influenza viruses (50 μl) were mixed an equal volume of 1 % chicken red blood cells (cRBCs) (Innovative Research, MI, USA) and the mixtures were incubated at 4 °C for 1 h for hemagglutination. The HA units of influenza virus were calculated as the highest dilutions that yielded complete hemaggutination. Hemagglutination inhibition (HI) assay was performed to test whether the extracts inhibit the receptor binding the viral HA to the cellular receptor. In V-bottom 96-well plates, two-fold serial dilutions of the extracts (25 μl) were incubated with 4 HA units of influenza viruses (25 μl) at 37 °C for 30 min. After the incubation, 50 μl of 1 % cRBCs were added to the wells and incubated at 4 °C for 1 h. HI concentrations of the extracts were determined as the lowest concentrations that completely inhibited virus-mediated hemagglutination.

### 2.6 Hemolysis inhibition assay

Two-fold serial dilutions of each extract (100 μl) were incubated with 3 × 10^4^ plaque forming units (PFUs) of A/PR8 (H1N1) virus (100 μl) for 30 min at 37 °C. After the incubation, 2 % cRBCs (200 μl) was added to the wells, followed by an additional incubation at 37 °C for 30 min and centrifuged at 4 °C for 8 min. After removing the supernatants, 450 μl of PBS was added to resuspend the pellets. Subsequently, 5 μl of 1 M acetic acid was added to lower the pH to 5.2, followed by incubation at 37 °C for 30 min. Centrifugation at 4 °C for 10 min was performed to precipitate cRBCs debris, and the absorbance of the supernatants was measured at 540 nm to evaluate the hemoglobin concentrations. Hemolysis inhibition was calculated as [(OD_virus_-OD_PBS_) – (OD_sample_ – OD_PBS_)]/(OD_virus_ – OD_PBS_) x 100 %.

### 2.7 Neuraminidase inhibition assay

Influenza viral NA enzyme activity was measured by an enzyme-linked lectin-based NA assay. 96-well plates were coated with 100 μl of 50 μg/ml fetuin (Sigma-Aldrich, MO, USA). Two-fold serial dilutions of A/PR8 (H1N1) virus were transferred onto fetuin-coated plates and were incubated at 37 °C for 1 h. The plates were washed and supplemented with 100 μl of lectin (Sigma-Aldrich) and incubated for 1 h at room temperature. After washing, 100 μl of TMB solution (Thermo Scientific, MA, USA) was added to each well and the reaction was stopped after 5 min by the addition of 50 μl of 2 N H_2_SO_4_. The absorbance of the mixtures was measured at 450 nm. For neuraminidase inhibition (NI) assay, two-fold serial dilutions of sera were mixed with the predetermined titer of A/PR8 (H1N1) virus with NA activity corresponding to OD_450_ of 1 and incubated for 1 h at 37 °C. The mixtures were then transferred to fetuin-coated plates and subjected to the subsequent procedures to measure the NA activity.

### 2.8 Influenza virus plaque assay

Two-fold serial dilutions of the extracts (500 μl) were mixed with 1 × 10^4^ PFUs of A/MA81 (H5N2) virus at 37 °C for 30 min or 2 h, and the mixtures were inoculated into MDCK cells for viral titration by plaque assay. MDCK cells cultured in 12-well plates with a confluency of 100 % were washed twice with DPBS. The preincubated mixtures containing the extract and A/MA81 (H5N2) virus were added to MDCK cells and were incubated at room temperature for 45 min on a rocker. After the incubation, the mixtures were aspirated, and overlay media containing 1 % low-melting agarose (Lonza, ME, USA) and 2.5 % of trypsin (Gibco) in Dulbecco's Modified Eagle Medium (Gibco) was added to the wells. After the overlays turned into solid, the plates were move into a CO_2_ incubator and were cultured at 37 °C for 2 or 3 days until viral plaques were formed. 4 % formaldehyde solution was added to each well for fixation of the cells. After the fixation, the overlay media was removed, and crystal violet solution was added to the wells to visualize the plaques.

### 2.9 GFP fluorescence analysis

To analyze dynamics of influenza virus infection, A/PR8-GFP reporter virus encoding GFP gene in the NS segment [18] was used. MDCK cells cultured in 96-well plates were infected with 100 PFUs/well of A/PR8-GFP virus in the presence or absence of SCH extracts. 24 h after the viral infection, the cells were washed with DPBS and stained with Hoechst 33342 for 15 min. The plates were analyzed using a Cytation 1 fluorescence microscope (BioTeK, TX, USA).

### 2.10 Statistical analysis

All the experiments were performed in triplicate and the data were expressed as the mean ± standard deviation (SD). Student's *t*-test was used to compare two different groups. The difference was considered statistically significant when *P* < 0.05 (∗∗∗; *P* < 0.001; ∗∗; *P* < 0.01; ∗; *P* < 0.05). Nonlinear regression analysis was done using GraphPad Prism version 9 software.

## 3 Results

### 3.1 *In vitro* cytotoxicity, polyphenol amounts, and antioxidative activity of SCH extracts

**Fig. 1:**
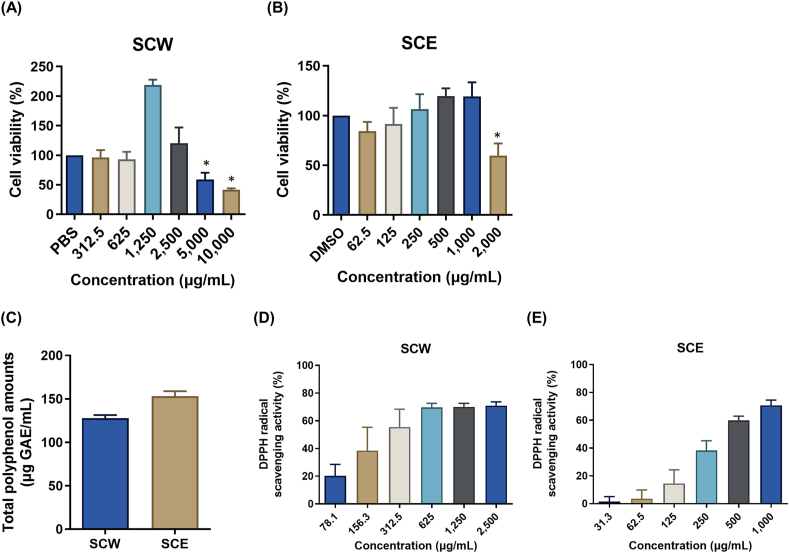
*In vitro* cytotoxicity, total polyphenol amount, and antioxidative activity of SCH extracts. (A and B) *In vitro* cytotoxicity of SCH extracts. Two-fold serial dilutions of the extracts were treated to MDCK cells for 24 h at 37 °C, with MEM and 2 % DMSO used as vehicle controls, and cell viability was measured using CCK-8 reagent. The cell viability after treatment of SCW (A) and SCE (B) are shown. (C) Total polyphenol amount contained in SCH extracts were measured by Folin-Ciocalteu method, with gallic acid as the reference standard. Total polyphenols contained in the extracts (1,000 μg/ml) was expressed as the gallic acid equivalent (GAE/ml). (D and E) Antioxidant activity of the extracts. Antioxidative activity of the extracts was measured using DPPH radical scavenging assay. Two-fold serial dilutions of each extract (20 μl) were mixed with 100 μM DPPH reagent (180 μl) and the mixtures were incubated for 30 min. After the incubation, the absorbance of the mixtures was measured at 514 nm. The DPPH radical scavenging activity of SCW (D) and SCE (E) are shown.

To examine *in vitro* cytotoxicity of SCH extracts, MDCK cell was treated with two-fold serial dilutions of each extract for 24 h at 37 °C, and cell viability was measured by CCK-8 assay. As compared to the control, SCH water extract (SCW) showed cytotoxicity at the concentration of 5,000 μg/ml and more and no cytotoxicity was observed at the concentrations of 2,500 μg/ml and less (Fig. 1A). SCH ethanol extract (SCE) was cytotoxic at 2,000 μg/ml but not cytotoxic at 1,000 μg/ml and less (Fig. 1B). It is well-known that plant-derived polyphenols have antioxidative activity. Total polyphenols and DPPH radical scavenging activity of the extracts were measured. 1,000 μg/ml of SCW contained polyphenols of 127.7 μg GAE/ml and 1,000 μg/ml of SCE contained polyphenols of 153.2 μg GAE/ml (Fig. 1C). DPPH radical scavenging activity of SCW was 20 %–70 % at 78.1 μg/ml ∼625 μg/ml, with an EC50 of 241.0 μg/ml (Fig. 1D). SCE demonstrated DPPH radical scavenging activity of 20 %–70 % at 125 μg/ml ∼1,000 μg/ml, with an EC50 value of 392.2 μg/ml (Fig. 1E). The subsequent experiments to elucidate the antiviral activities and molecular mechanisms were performed with the noncytotoxic concentrations of the extracts.

### 3.2 Virucidal activity of SCH extracts against influenza A virus

**Fig. 2:**
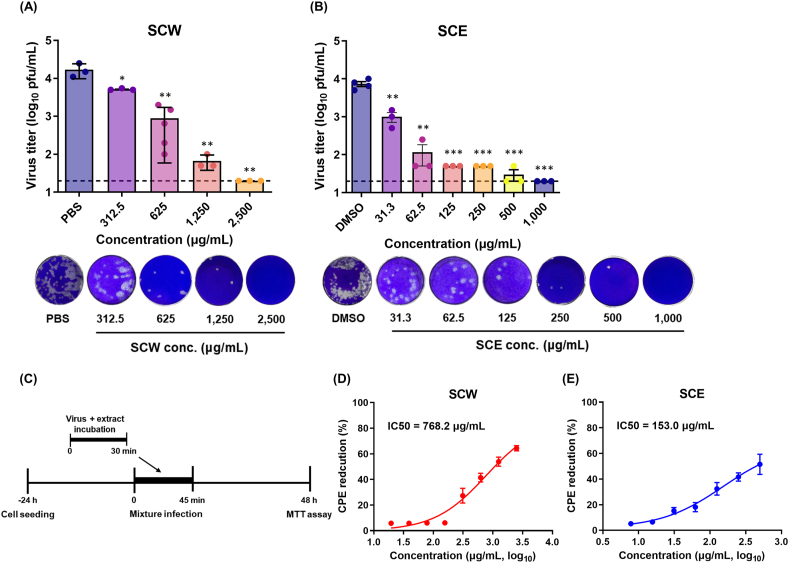
Virucidal activity of SCH extracts against influenza A virus. To determine the virucidal activity of the extracts, two-fold serial dilutions of the extracts were mixed with 10^4^ PFU of A/MA81 (H5N2) virus, and the mixtures were incubated for 30 min at 37 °C for viral inactivation. PBS and DMSO were used as negative controls. The mixtures were subjected to plaque assay on MDCK cells to measure the residual viral titers. The viral titers of the mixtures treated with SCW (A) and SCE (B) are shown with representative images of plaque assay of a dilution of 10^−1^. A dashed line indicates detection limit, 1.301. (C and D) CPE reduction by treatment of the extracts to A/MA81 (H5N2) virus. Two-fold serial dilutions of the extracts were treated to 0.01 MOI of A/MA81 (H5N2) virus for 30 min at 37 °C, and the mixtures were inoculated into MDCK cells cultured in 96-well plates. 48 h later, cell viability was measured to calculate the CPE reduction by the SCW (C) and SCE (D).

**Fig. 3:**
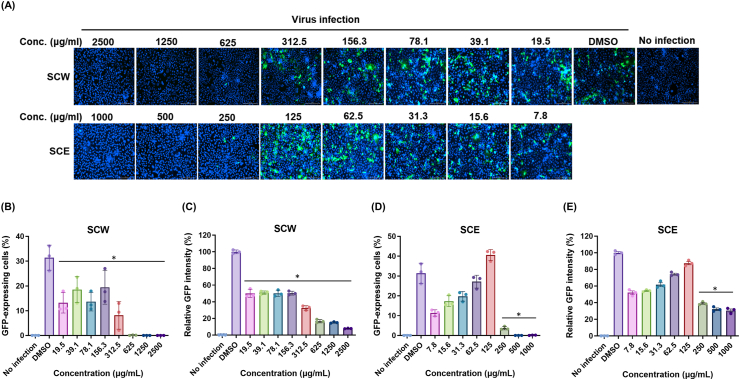
Fluorescence image analysis of virucidal activity of the SCH extracts. (A) Two-fold serial dilutions of SCW and SCE were treated to 1 MOI of A/PR8-GFP reporter virus and the mixtures were inoculated into MDCK cells cultured in 96-well plates. 24 h later, the cells were stained with Hoechst 33342 for staining of the nucleus of the cells, and GFP fluorescence image and GFP levels were detected by a fluorescence microscope. Quantitative fluorescence analysis was done in triplicate to estimate the GFP-expressing cells (B and D) and GFP intensity in each well (C and E).

We next examined whether the SCH extracts could inactivate influenza A virus. To investigate the virucidal activity of the extracts against influenza A virus, 1 × 10^4^ PFUs of A/MA81 (H5N2) virus was mixed with various concentrations of each extract for 30 min at 37 °C. After the incubation, the viral titers in the mixtures were measured by viral plaque assay. While treatment of PBS rarely reduced the viral titer, SCW completely removed the viral infectivity at 2,500 μg/ml, in which no viral plaques were visible (Fig. 2A). As the concentration of SCW decreased to 312.5 μg/ml, the residual viral titers increased to 5.2 × 10^3^ PFU/ml, but still demonstrating significant virucidal activity as compared to the control (Fig. 2A). SCE also demonstrated potent virucidal activity against influenza A virus. SCE showed complete virucidal activity at 1,000 μg/ml and the virucidal activity remained robust at the concentration of 31.3 μg/ml, demonstrating virucidal activity of approximately 86.4 % (Fig. 2B). The virucidal activity of SCW and SCE was also measured by cytopathic effect (CPE) after the viral infection pretreated with SCW or SCE (Fig. 2C). The CPE reduction by pretreatment of SCW to the virus was 64.3 %–5.8 % at 2,500 μg/ml ∼19.5 μg/ml, with an IC50 of 768.2 μg/ml (Fig. 2D). The CPE reduction of pretreatment of SCE to the virus was 51.5 %–5.2 % at 500 μg/ml ∼7.8 μg/ml, with an IC50 of 153.0 μg/ml (Fig. 2E). The virucidal activity of the extracts was also examined using A/PR8-GFP reporter virus that expresses GFP in the infected cells, allowing quantitative analysis of virus-infected cells. Fluorescence analysis indicated that while pretreatment of SCW to the virus at 625 μg/ml ∼2,500 μg/ml completely eliminated the viral infectivity, as shown by no GFP expression in the cells (Fig. 3A). Pretreatment of SCW to the virus at less than 312.5 μg/ml exhibited partial virucidal activity, permitting the residual viruses to replicate in the cells (Fig. 3A). Pretreatment of SCE to the virus at 500 μg/ml ∼1,000 μg/ml fully abrogated GFP expression, demonstrating complete virucidal activity, and the residual viruses were able to replicate in the cells at the lower concentrations (Fig. 3A). Quantitative fluorescence analysis revealed that GFP-expressing cells and the relative GFP intensity increased as SCW and SCE concentrations decreased (Fig. 3B ∼ 3E), suggesting that the virucidal activity of SCW and SCE operated in a dose-dependent manner. These results together suggest that SCH extracts possess virucidal activity against influenza A virus at noncytotoxic concentrations.

### 3.3 Inhibition activity of SCW and SCE on the viral entry into cells

**Fig. 4:**
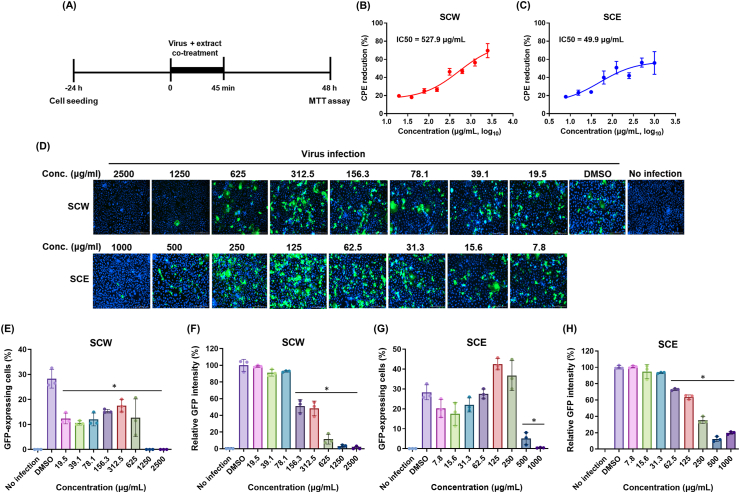
Inhibitory activity of SCH extracts on the viral entry into cells. (A and B) CPE reduction by co-treatment of the extracts and A/MA81 (H5N2) virus. Two-fold serial dilutions of the extracts and 0.01 MOI of A/MA81 (H5N2) virus were co-treated to cells cultured in 96-well plates for viral infection. 48 h later, cell viability was measured to estimate the CPE reduction by SCW (A) and SCE (B). (C) 1 MOI of A/PR8-GFP reporter virus and the various concentrations of the extract were co-treated to cells for viral infection. 24 h later, the cells were stained with Hoechst 33342 and GFP fluorescence were detected by a fluorescence microscope. Quantitative fluorescence analysis was done in triplicate to estimate the GFP-expressing cells (D and F) and GFP intensity in each well (E and G).

Next, we examined whether SCW and SCE inhibited the viral entry into cells. Firstly, CPE reduction was measured upon A/MA81 (H5N2) viral infection in the presence of various concentrations of SCW or SCE (Fig. 4A). Co-treatment of SCW and the virus to cells resulted in CPE reduction of 69.5 %–19.6 % at 2,500 μg/ml ∼19.5 μg/ml, with an IC50 of 527.9 μg/ml (Fig. 4B). Similarly, co-treatment of SCE and the virus to cells caused CPE reduction of 55.9 %–18.6 % at 1,000 μg/ml ∼7.8 μg/ml, with an IC50 of 49.9 μg/ml (Fig. 4C). The results suggest that SCW and SCE inhibit the viral entry into cells in a dose-dependent manner. Fluorescence image analysis using A/PR8-GFP reporter virus also demonstrated the inhibition activity of SCW and SCE on the viral entry into cells. Co-treatment of SCW and virus at more than 1,250 μg/ml completely suppressed GFP expression indicating no viral entry into cells, and at concentrations less than 625 μg/ml of SCW, the viruses were able to penetrate into cells as shown by GFP expression (Fig. 4D). Co-treatment of SCE and the virus also prevented the viral entry into cells at 1,000 μg/ml (Fig. 4D). Co-treatment of SCW and the virus at 625 μg/ml ∼2,500 μg/ml significantly reduced GFP-expressing cells and GFP intensity, also suggesting that SCW inhibited the viral entry into cells (Fig. 4E and F). Likewise, co-treatment of SCE and the virus at 500 μg/ml ∼1,000 μg/ml significantly reduced GFP-expressing cells and GFP intensity (Fig. 4G and H). These results together show that SCW and SCE possess inhibitory activity on the viral entry into cells.

### 3.4 Inhibition activity of SCH extracts on the biological functions of the viral HA

**Fig. 5:**
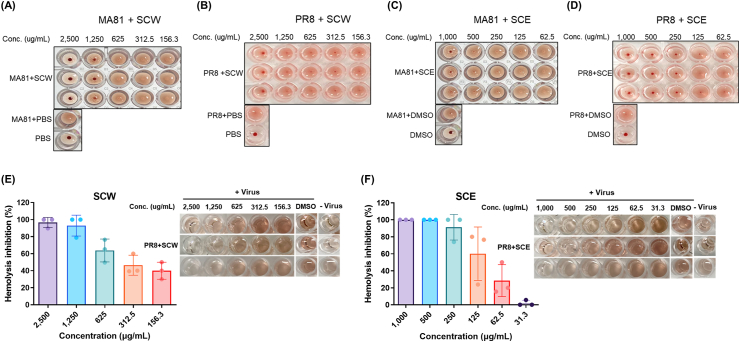
Inhibition of biological functions of viral HA by SCH extracts. (A to D) Hemagglutinin inhibition (HI) assay with SCH extracts. Four HA units (approximately 10,000 PFUs) of influenza A virus (25 μl) was incubated with two-fold serial dilutions of each extract (25 μl) for 30 min at 37 °C. After the incubation, 1 % of cRBCs (50 μl) were added to the mixture and were incubated for 1 h at 4 °C for hemagglutination. HI activity of SCW against A/MA81 (H5N2) (A) and A/PR8 (H1N1) (B) and HI activity of SCE against A/MA81 (H5N2) (C) and A/PR8 (H1N1) (D) are shown. (E and F) Hemolysis inhibition activity of SCH extracts. 3 × 10^4^ PFUs of A/PR8 (H1N1) virus was incubated with two-fold serial dilutions of each extract for 30 min at 37 °C. After the incubation, 2 % cRBCs (200 μl) were added to the mixtures and were further incubated at 37 °C for 30 min for hemagglutination. The mixtures were centrifuged to remove supernatants containing virus particles that did not bind to cRBCs. The pellet of viruses with cRBCs complex were resuspended in PBS (450 μl), and predetermined amount of 1 M acetic acid was added to adjust the pH to 5.2, and the mixtures were incubated at 37 °C for 30 min for hemolysis. After the incubation, the mixtures were centrifuged to pellet the cRBC debris, and the absorbance of supernatants was measured at 540 nm. Hemolysis inhibition (%) by SCW (E) and SCE are shown with the images of experimental results performed in triplicate.

Influenza virus HA proteins initiate infection cycle by binding to the sialic acid residues at the terminal end of glycoproteins, attaching virions to cell surface. In addition, HA proteins mediate the fusion event between the endosomal and viral membrane and lead to the release of viral genome into the cytoplasm. Thus, influenza viral HA inhibitors could serve as promising antiviral agents that can block the earlier stages of the viral infection life cycle, including the viral entry and the membrane fusion events. Hemagglutination inhibition (HI) assay was performed to test whether the extracts block receptor binding of influenza viral HA proteins. HI assay results show that SCW blocked A/MA81 (H5N2) viral HA binding to the sialic acid of cRBCs at concentrations more than 1,250 μg/ml (Fig. 5A). SCW also demonstrated HI activity against A/PR8 (H1N1) virus at 2500 μg/ml but not at lower concentrations (Fig. 5B). SCE also prevented A/MA81 (H5N2) viral HA-mediated receptor binding at 1,000 μg/ml (Fig. 5C). Against A/PR8 (H1N1) virus, SCE showed HI activity at more than 250 μg/ml (Fig. 5D). Thus, SCE demonstrated more potent HI activity against both A/MA81 (H5N2) and A/PR8 (H1N1) viruses than SCW. These results show that SCW and SCE have inhibitory activity on influenza viral HA-mediated receptor binding against both A/H1N1 and A/H5N2 viruses. We then examined whether the extracts inhibited the viral HA-mediated membrane fusion using hemolysis inhibition assay. SCW showed hemolysis inhibition of 96.5 %–40.0 % at 2,500 μg/ml ∼156.3 μg/ml against A/PR8 (H1N1) virus (Fig. 5E). SCE also exhibited hemolysis inhibition of 100 %–1.9 % at 1,000 μg/ml ∼31.3 μg/ml against the virus (Fig. 5F). Considering that hemolysis can occurs only after the viral HA binds to the sialic acid receptors on cRBCs, hemolysis inhibition by SCW and SCE can be attributed to HI activity of the extracts as described above (Fig. 5A ∼ 5D). However, it should also be noted that the viral titers used in hemolysis inhibition assay (approximately 3 × 10^4^ PFUs) are approximately 4-folds higher than those used in HI assay (approximately 10,000 PFUs), and that the extracts demonstrated hemolysis inhibition activity at lower concentrations than HI assay. For instance, while SCW did not exhibit HI activity against approximately 10,000 PFUs of A/PR8 (H1N1) virus at less than 1,250 μg/ml (Fig. 5B), SCW showed apparent hemolysis inhibition against approximately 40,000 PFUs of the virus at 1,250 μg/ml ∼156.3 μg/ml (Fig. 5E). The similar hemolysis inhibition activity was shown in SCE, in which SCE inhibited hemolysis at the concentrations lower than HI assay (Fig. 5D and F). These results suggest that SCW and SCE possess hemolysis inhibition activity, independent of HI activity.

### 3.5 Inhibitory activity of SCH extracts on the later stage of the viral life cycle and the NA activity

**Fig. 6:**
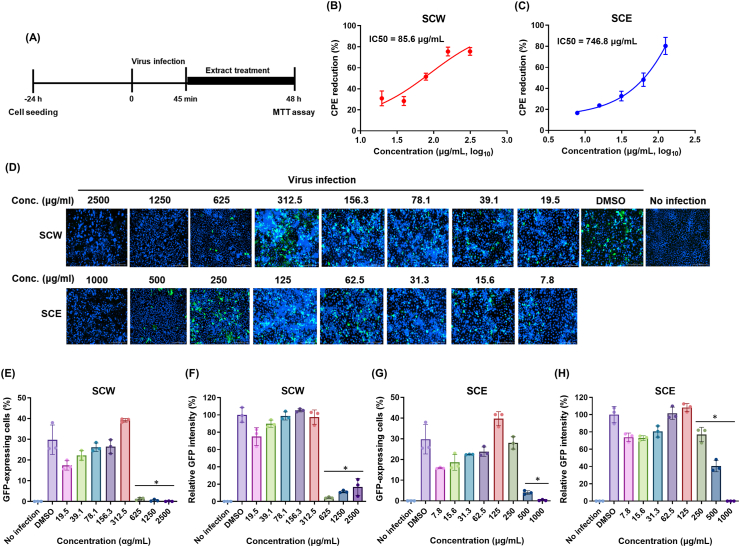
Inhibitory activity of SCH extracts on the later stage of influenza virus life cycle. (A and B) CPE reduction by posttreatment of the SCH extracts. MDCK cells cultured in 96-well plates were infected with 0.01 MOI of A/MA81 (H5N2) virus, and various concentrations of SCW or SCE was treated to the virus-infected cells. 48 h later, cell viability was measured to estimate CPE reduction by SCW (A) or SCE (B). (C) MDCK cells cultured in 96-well plated were infected with 1 MOI of A/PR8-GFP reporter virus and various concentrations of the extract was treated to the cells. 24 h later, the cells were stained with Hoechst 33342 and GFP fluorescence were detected by a fluorescence microscope. Quantitative fluorescence analysis was done in triplicate to estimate the GFP-expressing cells (D and F) and GFP intensity in each well (E and G).

**Fig. 7:**
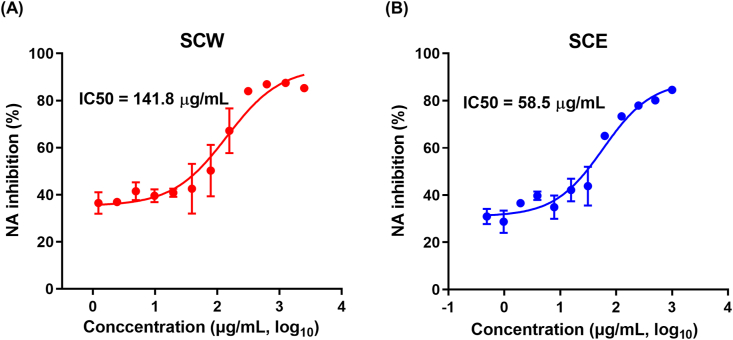
NA inhibition activity of SCH extracts. Enzyme-linked lectin-based assay was used to examine the inhibitory effects of the extracts on viral NA enzyme activity. Predetermined titer of A/PR8 (H1N1) virus (120 μl) was incubated with two-fold serial dilutions of each extract (120 μl) at 37 °C for 30 min. After the incubation, the mixtures were transferred to 96-well immunoplates pre-coated with fetuin and further incubated at 37 °C for 1 h. After incubation, the mixtures were aspirated, and 100 μl of lectin was dispensed into each well, followed by incubation at room temperature for 1 h. The remaining lectin on the plates were aspirated, and 100 μl of TMB was dispensed into each well. 5 min later, 50 μl of 2 N H_2_SO_4_ was dispensed into each well to stop the reaction, and absorbance was measured at 450 nm. NA inhibition (%) by SCW (A) or SCE (B) are shown. Nonlinear regression was analyzed using GraphPad Prism software.

To test whether SCW and SCE have inhibitory activity on the later stages the virus life cycle, MDCK cells were infected with A/MA81 (H5N2) virus, and SCW or SCE was post-treated to the cells until CPE was observed (Fig. 6A). Post-treatment of SCW resulted in CPE reduction of 75.6 %–30.9 % at 312.5 μg/ml ∼19.5 μg/ml, with an IC50 of 85.6 μg/ml (Fig. 6B). Similarly, post-treatment of SCE caused CEP reduction in a dose-dependent manner, with an IC50 of 746.8 μg/ml (Fig. 6C). The results suggest that SCW and SCE suppressed the viral replication when the extracts were post-treated to the virus-infected cells. Fluorescence image analysis using A/PR8-GFP reporter virus also substantiated the inhibition of the viral replication by post-treatment of SCW or SCE. Post-treatment of SCW significantly suppressed GFP expression at 625 μg/ml ∼2,500 μg/ml, and SCE also inhibited GFP expression at 500 μg/ml ∼1,000 μg/ml (Fig. 6D). The quantitative analysis of GFP-expressing cells and GFP intensity also indicated the inhibitory activity of SCW and SCE at 625 μg/ml ∼2,500 μg/ml and 500 μg/ml ∼1,000 μg/ml, respectively (Fig. 6E to H). Taken together, it is clearly suggested that SCW and SCE have inhibitory activity on the later stages of influenza virus life cycle. Considering that post-treatment of the extracts to the virus-infected cells may exert complex effects on viral replication by affecting multiple stages of the viral infection cycle, including the viral entry and release steps, the specific inhibitory activity of the extracts against influenza NA proteins was further analyzed. Influenza viral NA proteins have a sialidase activity that cleave off the sialic acids from cellular receptors of HA, enabling progeny virus particles to escape from infected cells at the later stage. Therefore, the enzyme activity of NA proteins has been an attractive target for designing antiviral drugs, as shown by the oseltamivir, the most widely used anti-influenza drug. We investigated whether the extracts blocked influenza viral NA enzyme activity using a neuraminidase inhibition (NI) assay. SCW showed NI activity of 87.5 %–36.5 % at 2,500 μg/ml ∼1.2 μg/ml, with an IC50 of 141.8 μg/ml (Fig. 7A). SCE also showed NI activity of 84.5 %–28.8 % at 1,000 μg/ml ∼0.5 μg/ml, with an IC50 of 58.5 μg/ml (Fig. 7B). These results together suggest that SCH extracts have inhibitory activity on the later stages of the viral life cycle, which may be due to inhibition of the viral NA enzyme activity.

## 4 Discussion

There is a substantial amount of literature addressing the antiviral activity of plant-derived natural products against diverse viruses including influenza viruses. However, very little has been known about the antiviral activity of SCH extracts against influenza viruses. To the best of our knowledge, our study is the first report demonstrating the antiviral activities of SCH extracts against influenza A viruses. In this study, SCH extracts demonstrated multifaceted antiviral activities, including virucidal activity, HI activity, HA-mediated hemolysis inhibition, and NI activity. Previous studies have reported the virucidal activity of the extracts or natural compounds derived from plants against influenza viruses. Studies showed that green tea extracts and catechin epigallocatechin-3-gallate crosslinked with influenza viral proteins, resulting in viral inactivation [20,21]. Another study demonstrated that trans-δ-viniferin derivatives isolated from plants exhibited virucidal activity against influenza A virus [22]. In addition, apigenin, a natural compound isolated from plants, was reported to exert virucidal activity by inhibiting cell attachment and entry of influenza A virus [23]. Furthermore, other studies showed that the virucidal activity of natural compounds could be achieved by affecting the integrity of the viral membrane without inhibiting the viral surface proteins [24,25].

In this study, SCW and SCE showed virucidal activity against influenza A virus, which can be explained by the inhibition of biological functions of the viral HA, including receptor binding and membrane fusion, as confirmed by HI assay and hemolysis inhibition assay. In line with the results, CPE reduction and GFP fluorescence analysis suggested the inhibition activity of SCH extracts on the viral entry step. The results suggest that SCH extracts provide a promising resource for the identification of the viral HA inhibitors for the development of novel antiviral agents against influenza viruses. Identification of influenza HA inhibitors that block cell attachment and entry of influenza virus have been promising strategies for the development of effective antiviral drugs [26,27]. Additionally, HA-mediated membrane fusion is an attractive target for designing novel antiviral drugs against influenza A viruses [10,28]. Our data suggest that SCH extracts contain natural compounds with inhibitory activities on both receptor binding and membrane fusion by viral HA. Additionally, SCH extracts demonstrated inhibition of the viral NA enzyme activity. Influenza NA inhibitors are major antiviral treatments that are currently approved for clinical uses, such as oseltamivir and zanamivir [29]. However, efforts have been made to develop novel NA inhibitors to overcome problems associated with the preexisting antiviral drugs, such as occurrence of resistant strains and side effects. Our data suggest that natural compounds contained in SCH extracts possess multiple antiviral activities targeting influenza viral surface proteins. Considering that influenza HA and NA play essential roles at the earlier and later stages of the viral infection cycle, SCH extracts provide promising resource for the development of novel HA or NA inhibitors.

Our present study has several limitations that should be addressed through further studies. First, the antiviral activity of SCH extracts against influenza viruses may result from cumulative effects of all the bioactive compounds contained in the extracts. Individual compounds responsible for eliciting antiviral activity should be identified through further delicate studies. It was previously shown that SCH contains various phytochemicals including polyphenols and flavonoids such as rutin, isoquercitrin, caffeoylquinic acid, dicaffeoylquinic acid, neosakuranin, chlorogenic acid, neochlorogenic acid, carotenoids, and ascorbic acid [16]. Among them, isoquercitrin and chlorogenic acid were reported to have antiviral activity against influenza viruses through direct effects on the viral targets. Isoquercitrin was reported to have antiviral activity against influenza A viruses via modulating HA and NA functions [30]. Chlorogenic acid was shown to exert antiviral activity against influenza A viruses by inhibition of NA enzyme activity [31]. Thus, it is worthwhile to investigate whether the other compounds have antiviral activity against influenza viruses and identify their precise antiviral mechanisms. Second, the present study focused on the antiviral activity of SCH extracts against the viral surface HA and NA proteins. Further studies are needed to examine the antiviral activity of SCH extracts in the virus-infected cells and their viral or host targets. Third, the antiviral activity of SCH extracts shown in the present study should be substantiated by *in vivo* animal studies for clinical relevance.

In summary, our present study demonstrated that SCH extracts contain bioactive natural compounds that have anti-influenza activity. The antiviral activity of the extracts targets influenza viral surface proteins including HA and NA, suggesting that HA or NA inhibitors could be newly identified in the extracts. Our results also merit further *in-vitro* studies to determine individual compounds with antiviral activity and the subsequent *in vivo* efficacy tests against influenza viruses for clinical relevance.

## Funding

This work was supported by a grant of the Korea Health Technology R&D Project through the Korea Health Industry Development Institute (KHIDI), funded by the 10.13039/501100003625Ministry of Health & Welfare, Republic of Korea (grant number: HI23C0673).

## CRediT authorship contribution statement

**Yewon Kim:** Investigation, Conceptualization. **Sanghyun Lee:** Methodology, Investigation. **Chulwoo Kim:** Writing – review & editing, Funding acquisition. **Sun-Woo Yoon:** Writing – review & editing, Funding acquisition. **Sejin Jeon:** Writing – review & editing, Methodology, Investigation. **Mi-Na Kweon:** Writing – review & editing, Methodology. **Baik-Lin Seong:** Writing – review & editing, Funding acquisition. **Sang-Uk Seo:** Writing – original draft, Supervision, Conceptualization. **Yo Han Jang:** Writing – review & editing, Writing – original draft, Investigation, Data curation, Conceptualization.

## Declaration of competing interest

The authors declare the following financial interests/personal relationships which may be considered as potential competing interests:Yo Han Jang reports financial support was provided by 10.13039/501100003625Ministry of Health & Welfare, Republic of Korea. If there are other authors, they declare that they have no known competing financial interests or personal relationships that could have appeared to influence the work reported in this paper.
